# Recent Trends in Nanoantioxidants

**DOI:** 10.3390/antiox14020207

**Published:** 2025-02-12

**Authors:** Matteo Perra, Maria Letizia Manca

**Affiliations:** Department of Life and Environmental Sciences, University of Cagliari, University Campus, S.P. Monserrato-Sestu Km 0.700, 09042 Monserrato, CA, Italy

The term “oxidative stress” refers to an imbalance between reactive oxygen species (ROS) generation and the antioxidant system, resulting in the increased formation of ROS and the reduced and/or inadequate efficiency of the physiological processes responsible for their elimination and homeostasis maintenance [1]. Oxidative stress contributes to cellular and molecular damage, including DNA mutations, lipid peroxidation, and protein denaturation, which are key mechanisms implicated in several chronic and non-chronic diseases such as cancer, heart disorders, and neurodegeneration [2,3]. Antioxidants play an essential role in counteracting oxidative stress by neutralizing ROS [4,5]. However, despite their importance, the therapeutic application of many antioxidant compounds, especially those derived from natural sources, faces significant challenges such as chemical instability, low solubility, rapid degradation in biological systems, and low permeability across cellular membranes, which limits their therapeutic effectiveness [6,7].

In recent years, nanotechnology has provided new platforms to overcome these issues. Nanoantioxidants, defined as nanoscale formulations of antioxidant compounds, have improved physicochemical properties, representing a cutting-edge approach to address the challenges of conventional antioxidants [8]. These include improved solubility, stability, and bioavailability, as well as the ability to control release and targeted delivery to specific tissues or cellular compartments. Such properties not only increase the efficiency of treatment but also reduce impacts outside the target. This paves the way for greater efficiency and accuracy, interfering with oxidative stress-related diseases [9,10].

Recent applications of nanoantioxidants are present in a wide range of fields, including oncology, dermatology, neuroprotection, and regenerative medicine [11,12,13,14]. These innovations highlight the transformative potential of nanoscale antioxidants in preventive and curative health care.

This Special Issue brings together groundbreaking studies at the intersection of nanotechnology and antioxidant research. It showcases different nanoantioxidant systems, developed to target oxidative stress in various biomedical applications, by taking advantage of nanoparticles unique characteristics, such as increased surface area, controlled release, and targeted delivery, and in turn reflecting the ever-changing landscape of nanoantioxidants research, showing the potential to revolutionize health care and improve patient outcomes.

The articles included in this collection highlight the importance of interdisciplinary collaboration that integrates biochemistry, materials science, and medicine to push the boundaries of medical innovation. They do not just address pressing health concerns but also pave the way for the development of personalized medicine strategies.

The majority of the antioxidant compounds are plant-derived phenolic compounds that are extensively used in traditional and modern medicine. In recent years, Houttuynia cordata has received increasing attention due to its potential antiviral activity. Moorthy et al. (Contribution 1), presented a captivating study on Houttuynia cordata extract (HCE) and its application in the synthesis of silver nanoparticles (AgNPs) through microwave-assisted processes. The research highlights HCE antioxidant properties and its role as a bio-reducing agent, resulting in the creation of highly stable and bioactive AgNPs. These nanoparticles demonstrated superior antimicrobial activity and enhanced biosafety, making them promising candidates for addressing antibiotic-resistant bacterial strains.

Hipólito et al. (Contribution 2), explored the metabolic profiling of cysteine pathways in non-small-cell lung carcinoma (NSCLC). Their findings reveal the differential reliance of NSCLC cell lines on hydrogen sulfide (H2S)-producing enzymes, offering insights into potential targets for personalized cancer therapies. By analyzing cysteine catabolism and employing selenium–chrysin compounds, the study demonstrated new avenues for metabolic interventions, paving the way for tailored treatment approaches.

Cytokine storm syndrome (CRS) and ROS overproduction are always implicated in lung acute injury (ALI) and acute respiratory distress syndrome (ARDS). Jin et al. (Contribution 3) addressed the urgent need for effective therapies against ALI by developing stem cell membrane-cloaked naringin-loaded nanoparticles (CM@Nar-NPs). In an ALI mouse model, the obtained nanoparticles efficiently targeted inflamed lung tissues, scavenged reactive oxygen species (ROS), and modulated macrophage polarization towards anti-inflammatory phenotypes. The promising results of the study highlighted the potential of biomimetic delivery systems in mitigating inflammation and improving survival rates in ALI models.

Fullerene derivatives, known as fullerenols, are able to exert several beneficial properties, such as antioxidant, protection against UV- and gamma-irradiation, or antiviral effects. Borisenkova et al. (Contribution 4), investigated the self-assembly ability in aqueous solution of fullerenol C60(OH)36, along with their potential antioxidant and antiviral effect. This highly hydroxylated fullerene derivative demonstrated significant ROS scavenging activity, cytoprotection against UV-induced damage, and moderate antiviral efficacy against influenza A(H1N1) and A(H3N2). These effects suggest the applicability of fullerenol as a shield against UV radiation and in antiviral therapies. 

Xue et al. (Contribution 5) provided a comprehensive review of nanoantioxidants for skin-related conditions, emphasizing their role in treating psoriasis, atopic dermatitis, and UV-induced skin damage. By utilizing nanocarriers to enhance antioxidant penetration and efficacy, these studies mark significant progress in addressing oxidative stress-related skin diseases. The review highlights the potential of nanoantioxidants, which can help to achieve personalized, effective, and sustainable treatments for several skin disorders.

Collectively, these studies illustrate the diverse applications of antioxidants across biomedical fields, from combating infections and cancer to enhancing skincare and antiviral defenses. As research progresses, the integration of antioxidant strategies with nanotechnology and personalized medicine holds promise for more effective and targeted therapeutic solutions. 
